# Effect of Growth Orientation on the Standard Heat Treatment Microstructure of Nickel-Based Single-Crystal Superalloy DD6

**DOI:** 10.3390/ma19040800

**Published:** 2026-02-18

**Authors:** Zhenyu Yang, Xiaogong Liu, Ji Wang, Zhiqiang Yang, Songsong Hu, Jian Zhang, Yushi Luo, Shenglong Dai

**Affiliations:** 1 Science and Technology on Advanced High Temperature Structural Materials Laboratory, AECC Beijing Institute of Aeronautical Materials, Beijing 100095, China; 2 School of Materials Science and Engineering, Xiangtan University, Xiangtan 411105, China

**Keywords:** nickel-based single crystal superalloy, growth orientation, heat treatment, microstructure, seeding method

## Abstract

Using the seeding method, nickel-based single-crystal superalloy DD6 specimens with different growth orientations were prepared in a liquid metal cooling (LMC) directional solidification furnace. Subsequent standard heat treatment was carried out, and the influence of growth orientation on the microstructure of the (001) crystal plane of the alloy after heat treatment was investigated. Results show that with the increase in growth orientation deviation angle from the <001> orientation, the area fraction of residual eutectic content is reduced, the average size and volume of pore and γ′ strengthening phase increase, and the cubicity of the γ′ strengthening phase decreases. The growth orientation does not significantly affect the morphology of residual eutectic content or the morphology of the strengthening phase of the γ′ in the dendrite cores and interdendrite regions. However, the size uniformity of the γ′ strengthening phase in dendrite cores and the width of the γ matrix channels decrease as the growth orientation deviation angle increases.

## 1. Introduction

Nickel-based single-crystal superalloys have become the material of choice for manufacturing turbine blades in aero-engines due to their excellent high-temperature properties, such as high strength, good oxidation resistance, hot corrosion resistance, and superior fatigue performance [1]. The mechanical properties of nickel-based single-crystal superalloys exhibit significant anisotropy [2,3,4]. To achieve optimal service performance, it is generally required that the <001> crystallographic orientation aligns with the blade axis during the preparation of single-crystal blades [5,6,7,8]. For working blades, the blades are usually assembled vertically in the mold, and single crystals are obtained through either the grain selection method or the seed crystal method [9,10]. Given that the broader platform structure of guide vanes is more prone to solidification defects, an inclined mold assembly combined with the seeding method has been increasingly adopted in recent years [11,12]. Therefore, during the fabrication of advanced <001> oriented single-crystal working blades and guide vanes, the growth orientations of nickel-based single-crystal superalloys show obvious differences.

Nickel-based single-crystal superalloys exhibit non-equilibrium solidification characteristics during directional solidification for single-crystal blades. Elements such as Al and Ta segregate to the interdendritic regions, while Re, W, Mo, and Cr concentrate in the dendritic cores. This leads to the formation of coarse γ′ phases and (γ + γ′) eutectic structures in the interdendritic areas, with significant differences in the morphology and size of the γ′ phases between the dendritic and interdendritic regions [13,14]. Numerous studies have shown that the growth orientation of nickel-based single-crystal superalloys significantly affects their directional solidification process [15,16,17]. In terms of microstructural morphology, a <001> growth orientation results in uniformly distributed dendrites with a four-fold symmetric cross-shaped pattern, whereas a <011> orientation produces asymmetric dendrites aligned in rows, forming a distinctive “W”-shaped dendritic arrangement [18]. As the growth orientation deviation angle from the <001> direction increases, the average dendrite arm spacing tends to increase significantly [19,20]. Ma and Grafe [21] measured the distribution of solute elements within dendritic cells and found that the segregation path of alloying elements along the <100> direction is longer than that along the <011> direction, resulting in a smaller variation range of element concentrations in the <100> direction compared to the <011> direction. Furthermore, Zhao [22] observed in studies on the influence of crystal orientation on microstructure during solidification that the farther the growth orientation deviates from <001>, the larger the γ′ phase size becomes; conversely, deviation from the <011> orientation shows the opposite trend. To meet the aero-engine requirements for turbine inlet temperature, a large number of refractory elements such as Re and W are usually added to the alloy to improve its temperature-bearing capacity [23,24,25]. This further exacerbates microsegregation and microstructural inhomogeneity and easily induces the formation of TCP phases in high-temperature environments, which severely impairs the alloy performance [26,27]. Although homogenization heat treatment can alleviate microsegregation and improve microstructural uniformity, it cannot fully eliminate the effects of the solidification process on the alloy’s microstructure and properties [14,28,29]. To date, studies on the influence of growth orientation on the heat-treated microstructure of nickel-based single-crystal superalloys have rarely been reported.

We have demonstrated that growth orientation exerts a significant influence on the solidification process of the nickel-based single-crystal high-temperature alloy DD6 [5]. As a mature commercial alloy, the heat treatment process that components made from it undergo prior to use has been standardized as a fixed heat treatment procedure. Therefore, in this study, DD6 nickel-based single-crystal superalloy bars with different growth orientations were prepared by the seeding method and subjected to the standard heat treatment process. The effects of growth orientation on dendrite morphology and precipitate distribution and characteristics, as well as the morphology and content of solid solution micropores in the heat-treated microstructure of the (001) crystal plane, were systematically investigated. This work can provide theoretical and experimental support for improving the preparation of single-crystal guide vanes by inclined mold assembly combined with the seeding method.

## 2. Materials and Methods

**Figure 1:**
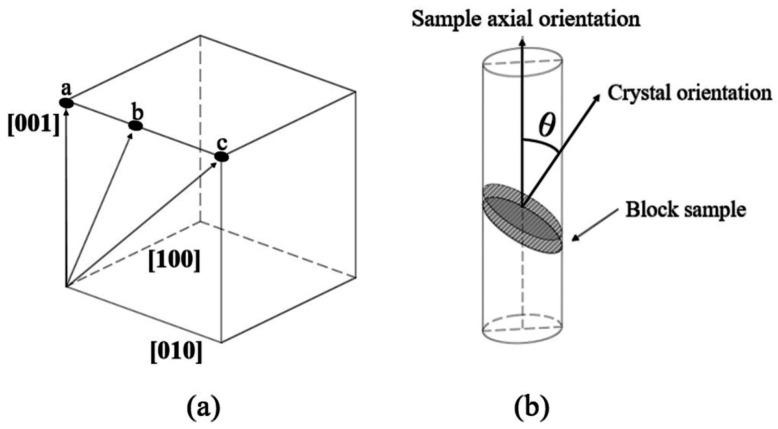
Schematic diagram of the orientation relationship between the seed and the single-crystal samples. (**a**) The orientation relationship of the seed axis (a, b, c) in the cubic crystal system. (**b**) Schematic diagrams of the relationship between the crystal orientation and the axial orientation of the single-crystal sample, as well as the relationship between the crystal orientation of the single-crystal sample and the single-crystal sample block.

The experimental material used in this study is the second-generation nickel-based single-crystal superalloy DD6, with its nominal chemical composition shown in Table 1. The crystal orientation of a large single-crystal bulk prepared by the grain selection method was calibrated using metallographic calibration. From this single-crystal bulk, three seed rods with different orientations were cut with dimensions of Φ7 mm × 20 mm. The crystal orientations of the seeds are labeled a, b, and c in Figure 1a. Among them, seed a is oriented along the <001> direction; seed b is oriented 20° away from the <001> direction; and seed c is oriented along the <011> direction. The single crystals were prepared in a liquid metal cooling (LMC) directional solidification furnace. An alumina crucible containing the seed and the DD6 master alloy bar was fixed onto the withdrawal rod of the directional solidification setup. During directional solidification, the system was maintained under vacuum (approximately 6 × 10^−2^ Pa). The furnace was heated to 1550 °C, held for 30 min, and then withdrawn downward at a speed of 25 µm/s over a distance of 70 mm. The relationship between the crystal orientation and the axial direction of the single crystal is illustrated in Figure 1b, where the angle θ between the crystal orientation and the axis is 0°, 20°, and 45°, respectively.

After directional solidification, the single-crystal bars were subjected to DD6 standard heat treatment following the schedule: 1290 °C/1 h + 1300 °C/2 h + 1315 °C/4 h/AC (air cooling) + 1120 °C/4 h/AC + 870 °C/32 h/AC [30]. Subsequently, a 7 mm thick specimen block with a (001) crystal plane was cut perpendicular to the <001> direction at 10 mm above the remelting interface, as shown in Figure 1b. The specimen block was sequentially mounted, ground, and polished, then etched with a corrosive agent composed of HNO_3_ (10 mL) + HF (20 mL) + C_3_H_6_O (30 mL) for 10 s. The etched specimens were used for subsequent microstructural analysis. The microstructure of the specimen cross-sections was observed and analyzed using a Leica-DM2700M optical microscope (Leica Microsystems, Wetzlar, Germany), an Apreo 2 field emission scanning electron microscope (FE-SEM) (Thermo Fisher Scientific, Waltham, MA, USA), and a matching energy-dispersive spectrometer (EDS). 

## 3. Results

### 3.1. Effect of Growth Orientations on Dendrites and Eutectic Content After Heat Treatment

**Figure 2:**
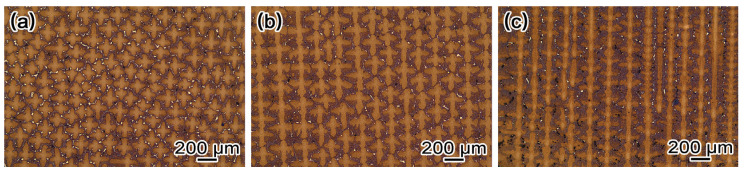
The dendrite morphology of the heat-treated microstructure under different growth orientations (**a**) <001>, (**b**) 20° deviation from the <001> direction, and (**c**) <011>.

Figure 2 displays the dendritic morphologies on the (001) crystal plane of single crystal specimens with different growth orientations after standard heat treatment. As can be observed, the dendritic morphology remains visible after heat treatment and exhibits significant differences across the various growth orientations, while substantial residual eutectic phases are still distributed in the interdendritic regions. When the growth orientation is aligned with the <001> orientation, the dendrites on the (001) plane display a uniformly distributed typical “cross-shaped” dendritic pattern. For a growth orientation deviated by 20° from the <001> orientation, the dendrites exhibit a linearly arranged bird-like configuration, where the secondary dendrite arms show asymmetry along the direction but remain symmetric along the direction. At the solidification along with <011> orientation, the dendritic morphology is similar to that observed at 20° deviation from the <001> direction, but the asymmetry of the secondary dendrite arms along the direction becomes more pronounced. The increase in asymmetry of secondary dendrite arms with deviation from the orientation can be attributed to the enhanced influence of the temperature gradient on the secondary arms facing the solidification interface. This promotes faster dissipation of solidification latent heat and consequently enhances their growth advantage [31].

**Figure 3:**
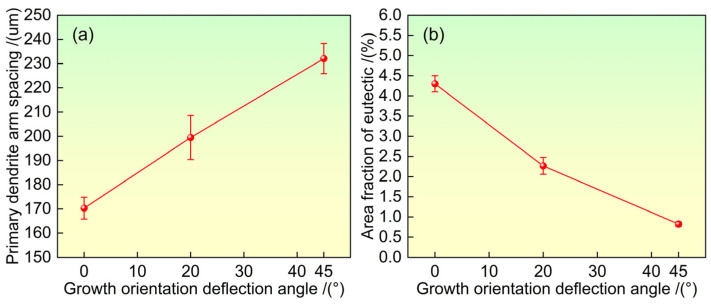
The influence of growth orientation on dendrite arm spacing (**a**) and residual eutectic content (**b**) after heat treatment.

Figure 3 shows the dendrite arm spacing and residual eutectic content under different growth orientations on the (001) plane. Under different growth orientations, the average primary dendrite spacing λ on the (001) crystal plane can be estimated using the following formula: λ = (A/N)^0.5^, where A is the cross-sectional area of the selected sample block, and N is the number of dendrite stems within the selected region. The calculation of residual eutectic content is obtained through the formula of A_0_/A_1_, where A_0_ represents the area of the residual eutectic phase, and A_1_ denotes the total tested area. It can be seen that as the growth orientation deviates further from the <001> direction, the average primary dendrite arm spacing increases, which is primarily due to changes in the solute field at the dendrite tip and the accelerated growth rate of secondary dendrite arms [32]. In contrast, the residual eutectic content gradually decreases with increasing deviation angle from the <001> orientation. As the deviation angle from the <001> orientation increases, the dendritic morphology of nickel-based single-crystal superalloy becomes increasingly complex and intertwined. This shortens the equivalent diffusion distance for elements while increasing the tortuosity of diffusion pathways, thereby promoting the diffusion and homogenization of solute elements during solidification [5].

### 3.2. Effect of Growth Orientations on γ′ Phase and γ Phase After Heat Treatment

**Figure 4:**
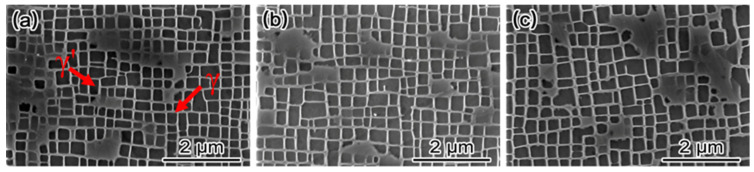
Morphologies of γ′ phase after heat treatment under different growth orientations. (**a**) <001> orientation, (**b**) 20° deviation from <001>, (**c**) <011> orientation.

The microstructure of single crystal superalloys is primarily composed of the γ matrix phase and the γ′ strengthening phase, which maintain a coherent relationship with each other. During directional solidification, solute redistribution leads to dendritic segregation, resulting in non-uniform morphology and size of the γ′ strengthening phase. Therefore, heat treatment is necessary to optimize the morphology, size, quantity, and distribution of the γ′ phase, thereby enabling the alloy to achieve its optimal mechanical performance. Figure 4 shows the morphology of the γ′ phase and γ phase within the dendrite stem regions after heat treatment under different growth orientations. In all orientations, the γ′ phase exhibits an approximately cuboidal shape. However, as the growth orientation deviates further from the <001> direction, the size of the γ′ phase gradually increases.

**Figure 5:**
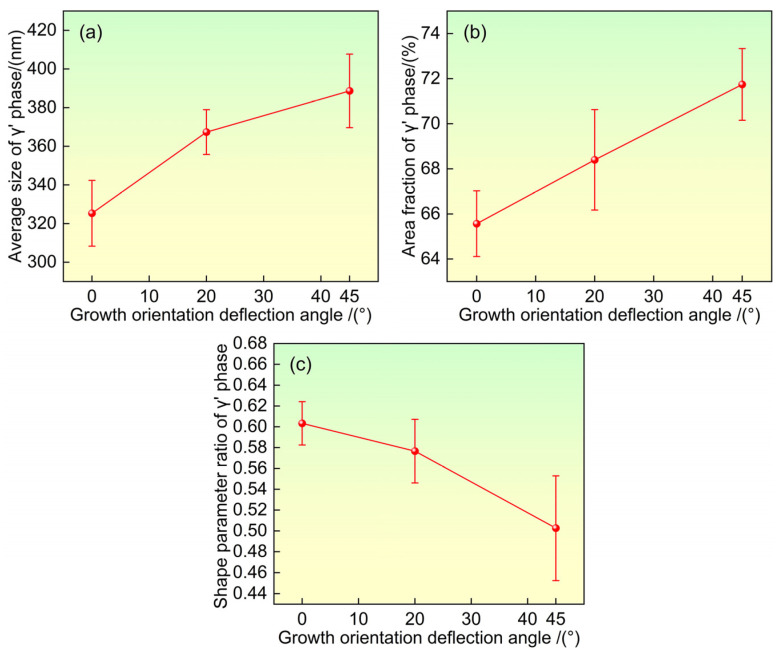
Sizes, area fractions, and shape parameter ratios of γ′ phase after heat treatment under different solidification directions. (**a**) The size of γ′ phase. (**b**) Area fraction of γ′ phase. (**c**) Shape parameter ratio of γ′ phase.

During the solution treatment stage of heat treatment, the coarse γ′ phase and the γ′/γ eutectic structure present in the as-cast state dissolve back into the γ matrix phase, while the γ′-forming elements are redistributed within the γ matrix. Upon entering the aging stage below the γ′ precipitation temperature, the high supersaturation of the γ matrix drives the γ′-forming elements to diffuse again into the γ′ precipitates, promoting their gradual growth. Once the parent γ solid solution is no longer supersaturated, further changes in the size of the γ′ phase are primarily governed by its coarsening behavior. At this stage, coarsening follows the Ostwald ripening mechanism, whereby larger γ′ precipitate particles grow further by absorbing surrounding smaller particles to reduce the overall interfacial energy. Figure 5a,b show the average size and area fraction of the γ′ strengthening phase after heat treatment under different growth orientations. It can be observed that as the angle between the growth orientation and the <001> direction increases, both the average size and the area fraction of the γ′ strengthening phase exhibit an increasing trend. In nickel-based superalloys, the growth and coarsening of the γ′ phase are diffusion-controlled processes, and its size evolution follows the LSW (Lifshitz–Slyozov–Wagner) coarsening equation [33]:

(1) $$r_{t}^{2}-r_{0}^{2}=Kt\;\;\;\;\;\;\;\;\;\;K=\left[\frac{8}{9}\frac{\Gamma V_{m}^{2}DC_{m}}{RT}\right]$$

In this equation, *r_t_* and *r*_0_ represent the average size of the γ′ strengthening phase at aging time t and at the onset of coarsening, respectively. *K* denotes the coarsening rate constant; *Γ* is the interfacial energy between the γ′ and γ phases; *V_m_* is the molar volume; *D* refers to the diffusion coefficient of elements between the γ′ and γ phases; *C_m_* is the solid solubility in the matrix phase; *R* is the gas constant; and *T* is the absolute temperature. An increase in the diffusion coefficient, an increase in the solid solubility of the γ matrix phase, and a decrease in the aging temperature all contribute to an increase in the size of the γ′ precipitates. According to experimental studies on the relationship between elemental segregation and growth orientation reported in the literature [34], as the growth orientation deviation angle increases, the segregation degree of various elements between the dendritic core and interdendritic regions decreases. This is consistent with our observation that the residual eutectic content decreases as the deviation angle increases. Specifically, with increasing deviation from the <001> orientation, the contents of γ′-forming elements such as Al and Ta in the dendritic core gradually rise, while the contents of γ matrix-forming elements such as Re, W, Cr, and Mo decline. Among these, elements like Re, Mo, and W not only have high diffusion activation energies but also large atomic radii, resulting in very low diffusion coefficients. They can slow down all processes controlled by elemental diffusion [35]. Consequently, at the growth orientation of <011> direction, the dendritic core contains fewer low-diffusivity elements compared to the <001> orientation, leading to a higher interdiffusion coefficient *D* between the γ′ and γ phases. Moreover, since the orientation exhibits the highest content of γ′-forming elements Al and Ta in the dendritic core, the γ matrix can dissolve more of these elements during solution heat treatment, meaning Cm is larger. In summary, under identical heat treatment conditions, as the angle between the solidification direction and the <001> orientation increases, the coarsening rate of the γ′ strengthening phase in the dendritic core accelerates, and its size increases accordingly. Under the same conditions, the reduced segregation of elements between the dendritic core and interdendritic regions with increasing deviation from the orientation leads to higher contents of γ′-forming elements Al and Ta in the dendritic core, which inevitably results in a corresponding increase in the area fraction of the γ′ phase.

**Figure 6:**
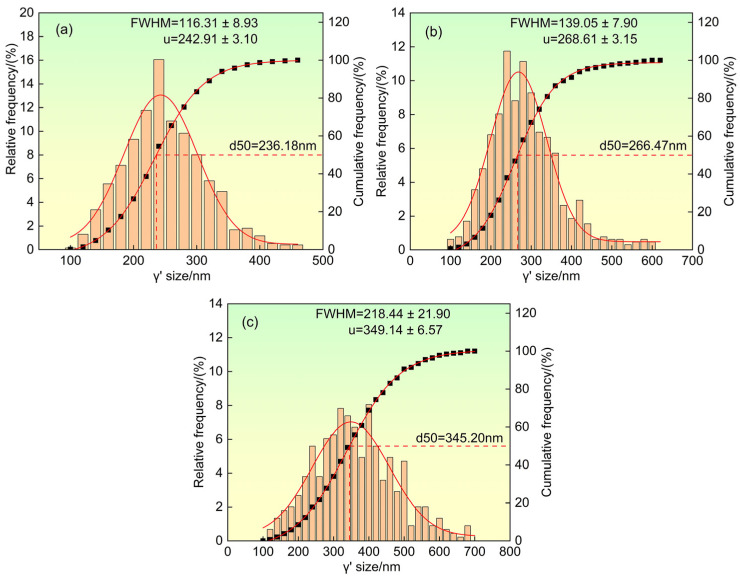
Size distribution of γ′ phase after heat treatment under different solidification directions. (**a**) <001> orientation, (**b**) deviation of 20° from <001>, and (**c**) <011> orientation, where FWHM denotes the full width at half maximum, and μ represents the mean value.

The higher the cubicity of the γ′ strengthening phase, the better the alloy strengthening effect. The shape parameter ratio η (the ratio of the shortest side length to the longest side length of the γ′ strengthening phase) well reflects this characteristic [36], with η ranging from 0 to 1. A value closer to 1 indicates better cubicity. Figure 5c shows the shape parameter ratio η of the γ′ strengthening phase in dendrite cores after heat treatment under different solidification orientations. As the growth orientation deviation angle from <001> orientation increases, the shape parameter ratio η decreases, indicating that the γ′ strengthening phase in the orientation has the worst cubicity. This is mainly due to the maximum diffusion and coarsening rate in dendrite cores of the orientation, which leads to the increase in its size, the decrease in the coherency between the γ′ phase and γ phase, and thus the decrease in the cubicity of the γ′ strengthening phase. Figure 6 shows the size distribution of the γ′ strengthening phase after heat treatment under different growth orientations. As the deviation angle from the direction increases, the size uniformity of the γ′ strengthening phase decreases.

**Figure 7:**
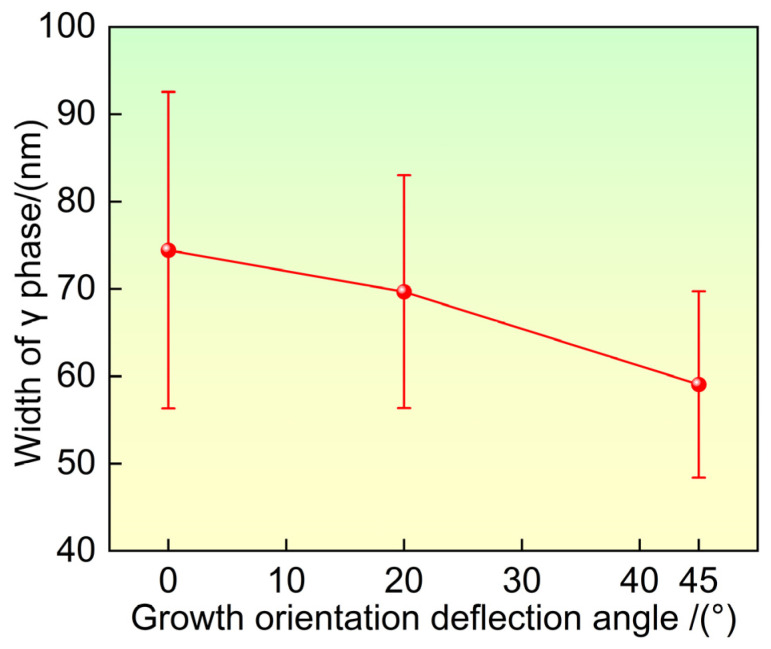
Width of the γ phase after heat treatment under different solidification directions.

The γ phase is a face-centered cubic (fcc) disordered solid solution of the Ni matrix. During the high temperature creep of nickel-based single-crystal superalloys, dislocations mainly move in the γ matrix. A narrower width of the γ matrix channel enhances its resistance to dislocation motion, thereby improving the creep performance of the alloy. Therefore, it is necessary to measure the width of the γ matrix channels after heat treatment under different growth orientations. The area method was used to measure the width of the γ matrix channels, with the formula as follows:

(2) $$\lambda =\sqrt{\frac{A}{N}}-\langle N\rangle$$

where *λ* is the width of the γ matrix channels; *A* is the area of the measured region; *N* is the number of γ′ strengthening phases in the measured region; and <*N*> is the average size of the γ′ strengthening phase. Figure 7 shows the width of the γ matrix channels after heat treatment under different growth orientations. As the deviation angle from the <001> direction increases, the width of the γ matrix channels narrows.

### 3.3. Effect of Growth Orientations on Porosity After Heat Treatment

**Figure 8:**
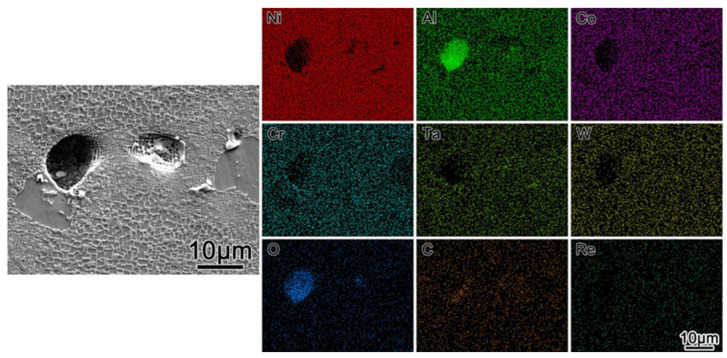
The elemental distribution in the pore regions within the (001) crystal plane was measured by EDS.

The solid solution micropores generated during heat treatment seriously affect the mechanical properties of the alloy. Studies have shown that the number of solid solution pores after heat treatment is more than five times that of solidification pores before heat treatment, and most of them are approximately spherical [37]. During the observation of the specimen microstructure, it was found that there seemed to be precipitates on the inner walls of the solid solution micropores. EDS analysis was performed on the solid solution micropores in this experiment, and the results are shown in Figure 8. The analysis shows that Al and O are highly enriched in the solid solution micropores. Considering that alumina polishing paste was used during the metallographic polishing process, it is possible that due to the large size of the solid solution micropores, the alumina polishing paste remains on the inner walls of the pores and is difficult to clean thoroughly, leading to the enrichment of Al and O in the EDS analysis.

**Figure 9:**
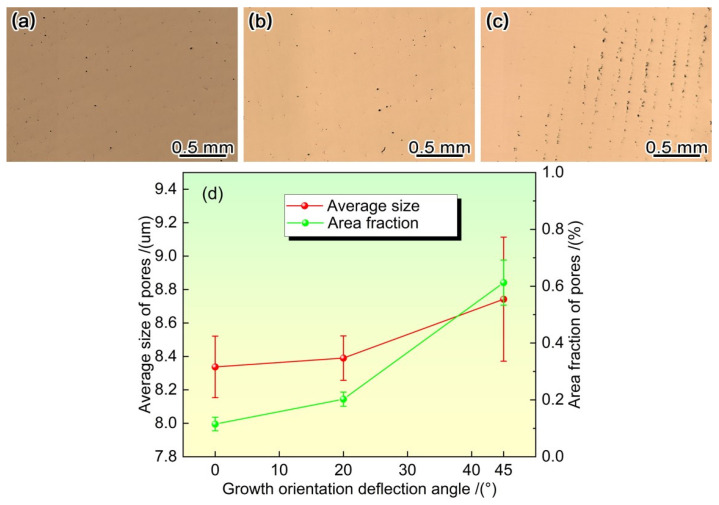
The morphology distribution and content of pores under different solidification orientations. (**a**–**c**) The morphology distribution of solution micro-holes under different growth orientations: (**a**) <001> orientation, (**b**) deviation of 20° from <001>, (**c**) <011> orientation, (**d**) the size and content of solution micro-holes under different growth orientations.

Figure 9a–c show the morphology and distribution of pores in the (001) crystal plane after heat treatment under different growth orientations. When the growth orientation is <001> direction, the solid solution micropores with high sphericity are uniformly and randomly distributed in the (001) crystal plane. When the growth orientation is <011> direction, a large number of solid solution micropores appear, which are unevenly distributed and arranged in a linear manner. This is mainly because during the solidification of the <011> orientation, the dendrites are linearly arranged in the direction, and the asymmetric secondary dendrites in the direction occupy the interdendritic regions, resulting in the distribution of solid solution micropores in the interdendritic regions on both sides of the linearly arranged dendrites in the direction, and ultimately leading to the linear arrangement of solid solution micropores.

Figure 9d shows the average size and volume of solid solution pores after heat treatment under different growth orientations. It can be seen that as the deviation angle from the <001> direction increases, both the size and content of solid solution micropores increase. We have previously reported that in the as-cast state, as the growth orientation deviation angle increases, the porosity content only slightly increases (from 0.1% to 0.05%) [5]. The formation of solid solution micropores is caused by the Kirkendall effect. During the solid solution heat treatment, elements such as Re and W enriched in dendrite cores diffuse to interdendritic regions, while elements such as Al and Ta enriched in interdendritic regions diffuse to dendrite cores. However, due to the much lower diffusion coefficients of elements such as Re and W compared to Al and Ta, this results in unbalanced diffusion between elements. The high diffusion flux of Al and Ta elements in interdendritic regions leads to a relatively high vacancy concentration in interdendritic regions, and the gradual aggregation of vacancies may form pores. Based on the Kirkendall effect, Bokstein [37] et al. established a model for the change in micropore area fraction during solid solution, as follows:

(3) $$V_{p}\left(t\right)-V_{p}^{0}=\frac{4}{27}\sum_{i}^{n}C_{i}^{e}\left(1-k_{i}^{0}\right)\left[1-exp\left(-27tD_{i}/2R^{2}\right)\right]$$

where *V_p_*(*t*) is the micropore area fraction at time *t* of solid solution heat treatment; $V_{p}^{0}$ is the area fraction of solidification micropores in the as-cast alloy; $C_{i}^{e}$ is the equilibrium concentration of element *i*; $k_{i}^{0}$ is the segregation coefficient of element *i* in the as-cast alloy; *R* is half of the primary dendrite arm spacing; and *D_i_* is the diffusion coefficient of element *i*. It can be seen that the area fraction of solid solution micropores is related to the solid solution heat treatment temperature, time, as-cast solidification pores, dendrite arm spacing, and segregation degree. The increase in their values will increase the area fraction of solid solution micropores. In the as-cast state, both the tortuous dendrite morphology and the decrease in eutectic area fraction will increase the pressure drop between dendrites, thereby increasing the area fraction of solidification micropores [38]. Therefore, when the solidification direction is <011> direction, the as-cast solidification micropores are larger than those in other orientations, and the larger dendrite arm spacing further enhances the unbalanced diffusion of elements between dendrite cores and interdendritic regions. For as-cast single crystals with different solidification orientations subjected to the same standard heat treatment, the larger the angle between the solidification direction and the growth orientation of <001> direction, the higher the area fraction of solid solution micropores.

## 4. Discussion

The present study demonstrates that the growth orientation exerts a profound and lasting influence on the heat-treated microstructure of a DD6 single-crystal superalloy, even after a standardized thermal processing route. This underscores that the solidification stage establishes a critical initial condition that predetermines, to a significant extent, the final microstructural state achievable upon heat treatment.

The experimental results collectively reveal a clear trend: with increasing deviation of the growth direction from the ideal <001> orientation, the heat-treated microstructure evolves towards a state characterized by larger and less uniform γ′ precipitates, reduced γ′ cubicity, narrower γ matrix channels, a lower fraction of residual eutectic content, and a notably higher density of solution micropores. These features are not independent but are intrinsically linked through the fundamental roles of elemental segregation and diffusion. The altered dendritic morphology associated with off-axis growth (Figure 2) modifies the local solute fields during solidification [19,20], leading to a distinct initial distribution of alloying elements, particularly the γ′-forming (Al, Ta) and γ-forming (Re, W) elements [5,34]. This inherited segregation pattern then governs the subsequent diffusion-controlled processes during heat treatment—namely γ′ precipitation/coarsening (as described by the LSW theory) and the unbalanced interdiffusion leading to pore formation via the Kirkendall effect.

The observed microstructural changes have direct, though complex, implications for the alloy’s high-temperature mechanical properties. The increase in γ′ size and the deterioration of its cubic morphology and size uniformity are generally considered detrimental to the alloy’s strength and stability [36]. Concurrently, the significant increase in solution porosity, which acts as a potent stress concentrator, would be expected to severely impair tensile ductility and fatigue resistance [37]. Conversely, the narrowing of the γ matrix channels, a feature observed with increasing orientation deviation, could potentially enhance resistance to dislocation glide and thus benefit creep performance. The net impact on service properties is therefore a complex interplay of these opposing factors. From a purely microstructural optimization perspective targeting fine, cubic, and uniform γ′ precipitates alongside minimal porosity, the traditional <001> growth orientation yields the most favorable microstructure in DD6 alloy after standard heat treatment. This finding provides a fundamental microstructural rationale for the stringent orientation control required in the manufacture of critical components like turbine blades.

It is noteworthy that conventional Time–Temperature–Transformation (TTT) diagrams, which typically assume a homogeneous initial state, may have limited predictive accuracy for such orientation-dependent outcomes. The reason lies in the non-uniform initial segregation profile, which is a function of growth orientation. The fixed heat treatment interacts with this spatially varying starting condition, leading to the differential microstructural evolution captured in this study. Therefore, accurate prediction requires coupling transformation kinetics with realistic initial micro-segregation models.

In summary, controlling growth orientation is critical not only for avoiding solidification defects but also for ensuring a superior and reproducible heat-treated microstructure that is the foundation of excellent service performance. To fully quantify the property implications and establish a definitive processing–structure–property relationship, future work must involve systematic mechanical testing. Specifically, tensile, creep, and fatigue properties along the <001> loading direction should be evaluated using specimens encompassing the characterized range of growth orientations, thereby directly linking the microstructural metrics presented here to macroscopic engineering performance.

## 5. Conclusions

(1) After standard heat treatment, microstructural inhomogeneity still exists in single crystal specimens with different growth orientations, and dendrite morphology and residual eutectic content are still visible on the (001) crystal plane. As the growth orientation deviation angle from the <001> direction increases, the dendrites on the (001) crystal plane transform from uniform arrangement to linear arrangement, and the dendrite asymmetry in the direction increases. The primary dendrite arm spacing increases with the increase in the deviation angle from the <001> direction, while the area fraction of residual eutectic content decreases.
(2) After heat treatment, the γ′ phase exhibits a regular approximately cubic shape under different growth orientations. As the deviation angle from the <001> direction increases, the size distribution uniformity of the γ′ phase deteriorates, and the average size and area fraction of the γ′ phase increase. The shape parameter ratio of the γ′ phase and the width of the γ matrix channels decrease with the increase in the deviation angle from the direction.
(3) After standard heat treatment, a large number of solid solution micropores appear between dendrites. As the deviation angle from the <001> direction increases, the distribution of solid solution micropores becomes increasingly uneven, and their content increases significantly. When the growth orientation is direction, the solid solution micropores exhibit a linear arrangement characteristic.

## Figures and Tables

**Table 1:** Nominal chemical composition of DD6 alloy (mass fraction/%) [5].

**Element**	Cr	Co	Mo	W	Ta	Re	Nb	Al	Hf	C	Ni
**Content**	4.3	9	2	8	7.5	2	0.5	5.6	0.1	0.006	Bal.

## Data Availability

The original contributions presented in the study are included in the article. Further inquiries can be directed to the corresponding author.

## References

[1] Reed R.C. (2008). The Superalloys: Fundamentals and Applications.

[2] Qin J., Yang W., Cui S., Liu C., Wang Q., Xin Z., Zhang Y., Su H., Liu L. (2025). The action mechanism of secondary orientation on creep life in 001 oriented Ni-based single-crystal blades. Mater. Res. Lett..

[3] Sakaguchi M., Mase K., Sasakura I., Tanaami S., Fukuda T., Karato T. (2026). Anisotropic fatigue limit estimation for a notched single crystal superalloy based on the theory of critical distances. Int. J. Fatigue.

[4] Guan Q.Y., Li Y.M., Wang X.G., Tan Z.H., Mu Y., Tao X.P., Zhang C.H., Zhang S., Liu J.D., Li J.G. (2025). Effect of orientation on the stress rupture properties of a Ru-containing fourth-generation single crystal superalloy at 1140 °C. Mater. Today Commun..

[5] Yang Z., Hu S., Chen Y., He M., Li W., Bai W., Wang X. (2025). Effect of Growth Orientation on Solidification Process of Nickel-Based Single Crystal Superalloy DD6. Metall. Mater. Trans. A.

[6] Wang B., Zeng L., Xia M., Ren N., Li J. (2022). Substrate stimulating technique for Ni-based single crystal superalloy preparation during direction solidification. Mater. Des..

[7] Zhang X.L., Liu Z.H., Liu G.Q., Zhou Y.Z., Meng X.B. (2022). A new mechanism of grain selection in spiral selector: The competitive growth between the entrance grains. Mater. Today Commun..

[8] Zhang C., Hu X., Yuan X., Ren W., Lu H., Ding B., Li Q., Zheng T., Lei Z., Zhong Y. (2024). Optimization of grain-selection behavior in the spiral selector during Cusp-magnetic-field-assisted directional solidification of single-crystal superalloy. J. Mater. Res. Technol..

[9] Wang F., Ma D., Mao Y., Bogner S., Buehrig-Polaczek A. (2016). Influence of the Size Effect on the Microstructures of the DWDS- and Bridgman-Solidified Single-Crystal CMSX-4 Superalloy. Metall. Mater. Trans. B Process Metall. Mater. Process. Sci..

[10] Liu Z.-F., Miao K., Lian W.-B., Lu Z.-L., Yi C., Li D.-C. (2021). Effect of mould baffle technology on stray grain formation in single crystal blades by integral fabrication based on 3D printing. China Foundry.

[11] Huo M., Liu L., Yang W.C. (2023). Dendrite Deformation in the Rejoined Platforms of Ni-Based Single-Crystal Superalloys. Adv. Eng. Mater..

[12] Yang Z.Y., Liu C.G., Hu S.S., Zheng S.J., Luo Y.S., Dai S.L. (2021). Influence of platform position on stray grain nucleation in Ni-based single-crystal dummy blade clusters. China Foundry.

[13] Caldwell E.C., Fela F.J., Fuchs G.E. (2004). The segregation of elements in high-refractory-content single-crystal nickel-based superalloys. JOM.

[14] Zhang H., Liu Y., Chen X., Zhang H., Li Y. (2017). Microstructural homogenization and high-temperature cyclic oxidation behavior of a Ni-based super alloywith high-Cr content. J. Alloys Compd..

[15] Zhao X., Liu L., Yu Z., Zhang W., Fu H. (2010). Microstructure development of different orientated nickel-base single crystal superalloy in directional solidification. Mater. Charact..

[16] Zhao X.B., Liu L., Yang C.B., Li Y.F., Zhang J., Li Y.L., Fu H.Z. (2011). Influence of crystal orientation on cellular growth of a nickel-base single crystal superalloy. J. Alloys Compd..

[17] Jiang W., Han D., Dong L., Li K., Meng X., Li Q. (2024). Influence of Crystal Orientation on Freckle Formation in Single Crystal Heavy-Plate Castings. Metall. Mater. Trans. A Phys. Metall. Mater. Sci..

[18] Yang C., Liu L., Zhao X., Li Y., Zhang J., Fu H. (2012). Dendrite morphology and evolution mechanism of nickel-based single crystal superalloys grown along the ⟨001⟩ and ⟨011⟩ orientations. Progress. Nat. Sci. Mater. Int..

[19] Hui X., Jian-Yuan W., Chang-Le C., Ke-Xin J., Li-Fei D. (2014). Dendrite to symmetry-broken dendrite transition in directional solidification of non-axially oriented crystals. Chin. Phys. B.

[20] Xing H., Dong X., Wang J., Jin K. (2018). Orientation Dependence of Columnar Dendritic Growth with Sidebranching Behaviors in Directional Solidification: Insights from Phase-Field Simulations. Metall. Mater. Trans. B Process Metall. Mater. Process. Sci..

[21] Ma D., Grafe U. (1999). Microsegregation in directionally solidified dendritic-cellular structure of superalloy CMSX-4. Mater. Sci. Eng. A.

[22] Zhao X.B., Liu L., Gao S.F., Ge B.M., Zhang J., Li Y.L., Fu H.Z. (2011). Effect of orientation on the microstructure of nickel-based crystalline superalloys. Cryst. Res. Technol..

[23] Tan X.P., Liu J.L., Jin T., Hu Z.Q., Hong H.U., Choi B.G., Kim I.S., Jo C.Y. (2012). Effect of ruthenium on tensile properties of a single crystal Ni-based superalloy. Met. Mater. Int..

[24] Guo H.Y., Tan Z.H., Li Y.M., Zou M.K., Tao Y., Wang X.G., Liu J.D., Liu J.L., Yang Y.H., Li J.G. (2024). Diffusion of alloying elements during high temperature oxidation in a low-cost third generation Ni-based single crystal superalloy. Mater. Charact..

[25] Zhou Y., Zhao X., Fan Y., Yue Q., Xia W., Pan Q., Cheng Y., Li W., Gu Y., Zhang Z. (2025). Composition Optimization in Alloy Design for Nickel-Based Single Crystal Superalloy: A Review. Metals.

[26] Duan H., Li G., Chen X., Ai C., Kang J., Ru Y., Zhang H., Pei Y., Li S., Gong S. (2025). Effect of Ru addition on γ/γ′ partitioning behavior of alloying elements, γ/γ′ lattice misfit and 1200 °C creep properties of Ni-based single-crystal superalloy. Intermetallics.

[27] Liu Y., Li Y., Yan Z., Yu D., Liang C., Xue M., Geng C., Su R., Li S., He L. (2025). Effects of dendritic segregation and local internal stress on microstructure evolution in a 3rd-generation single crystal superalloy during thermal exposure. Mater. Des..

[28] Paraschiv A., Matache G., Puscasu C. (2018). The effect of heat treatment on the homogenization of CMSX-4 Single-Crystal Ni-Based Superalloy. Transp. Res. Procedia.

[29] Strutt V.C.I., Jenkins B.M., Woolrich J.M., Appleton M., Moody M.P., Bagot P.A.J. (2023). Effect of microsegregation and heat treatment on localised γ and γ′ compositions in single crystal Ni-based superalloys. J. Alloys Compd..

[30] Li W., Zhao C.L., Zhang X., Wang Q., Li P., Fang X., Peng W.Y. (2023). Low cycle fatigue properties and fatigue mechanism of DD6 single crystal superalloy under asymmetrical cyclic loading. J. Aeronaut. Mater..

[31] Zhang L.F., Yang P., Zhao J.C., Zeng Q., Han F.K. (2011). Influence of the Solidification Directions on Dendritic Structures in a Single Crystal Superalloy. J. Mater. Eng..

[32] Grugel R.N., Zhou Y. (1989). Primary dendrite spacing and the effect of off-axis heat flow. Metall. Trans. A.

[33] Lifshitz I.M., Slyozov V.V. (1961). The kinetics of precipitation from supersaturated solid solutions. J. Phys. Chem. Solids.

[34] Zhao X., Liu L., Zhang W., Liu G., Zhang J., Fu H. (2009). Segregation behavior of alloying elements in different oriented single crystal nickel based superalloys. Mater. Lett..

[35] Shang Z., Wei X., Song D., Zou J., Liang S., Liu G., Nie L., Gong X. (2020). Microstructure and mechanical properties of a new nickel-based single crystal superalloy. J. Mater. Res. Technol..

[36] Van Sluytman J.S., Pollock T.M. (2012). Optimal precipitate shapes in nickel-base γ–γ′ alloys. Acta Mater..

[37] Bokstein B.S., Epishin A.I., Link T., Esin V.A., Rodin A.O., Svetlov I.L. (2007). Model for the porosity growth in single-crystal nickel-base superalloys during homogenization. Scr. Mater..

[38] Lecomte-Beckers J. (1988). Study of microporosity formation in nickel-base superalloys. Metall. Trans. A.

